# Attentive transformer deep learning algorithm for intrusion detection on IoT systems using automatic Xplainable feature selection

**DOI:** 10.1371/journal.pone.0286652

**Published:** 2023-10-16

**Authors:** Demóstenes Zegarra Rodríguez, Ogobuchi Daniel Okey, Siti Sarah Maidin, Ekikere Umoren Udo, João Henrique Kleinschmidt

**Affiliations:** 1 Department of Computer Science, Federal University of Lavras, Minas Gerais, Brazil; 2 Center for Engineering, Modeling, and Applied Social Sciences, Federal University of ABC, Santo Andre, São Paulo, Brazil; 3 Faculty of Data Science and Information Technology (FDSIT), INTI International University, Nilai, Malaysia; 4 Department of Electrical/Electronic Engineering, Michael Okpara University of Agriculture, Umudike, Nigeria; Vellore Institute of Technology: VIT University, INDIA

## Abstract

Recent years have witnessed an in-depth proliferation of the Internet of Things (IoT) and Industrial Internet of Things (IIoT) systems linked to Industry 4.0 technology. The increasing rate of IoT device usage is associated with rising security risks resulting from malicious network flows during data exchange between the connected devices. Various security threats have shown high adverse effects on the availability, functionality, and usability of the devices among which denial of service (DoS) and distributed denial of service (DDoS), which attempt to exhaust the capacity of the IoT network (gateway), thereby causing failure in the functionality of the system have been more pronounced. Various machine learning and deep learning algorithms have been used to propose intelligent intrusion detection systems (IDS) to mitigate the challenging effects of these network threats. One concern is that although deep learning algorithms have shown good accuracy results on tabular data, not all deep learning algorithms can perform well on tabular datasets, which happen to be the most commonly available format of datasets for machine learning tasks. Again, there is also the challenge of model explainability and feature selection, which affect model performance. In this regard, we propose a model for IDS that uses attentive mechanisms to automatically select salient features from a dataset to train the IDS model and provide explainable results, the TabNet-IDS. We implement the proposed model using the TabNet algorithm based on PyTorch which is a deep-learning framework. The results obtained show that the TabNet architecture can be used on tabular datasets for IoT security to achieve good results comparable to those of neural networks, reaching an accuracy of 97% on CIC-IDS2017, 95% on CSE-CICIDS2018 and 98% on CIC-DDoS2019 datasets.

## Introduction

The IoT network is a large network space that lets a lot of devices around the world connect to each other to disseminate information [1]. This reduces the amount of work that people have to do and makes the digital world easier to access, more productive, and easier to control. Because the IoT ecosystem is made up of many different types of devices, it is very vulnerable to cyberattacks that take advantage of the system’s weaknesses to stop it from working [2, 3]. Cyber threats are getting more complex and dangerous, and the IoT infrastructure is proliferating [4, 5]. This makes it very important to improve security in smart IoT networks. The authors of [6] identified and classified the security risks associated with any IoT system into four categories: risks relating to physical impacts on network devices; risks relating to the device and user authentication and validation; security concerns relating to violations of rules and the confidentiality, integrity, and availability (CIA) of information systems; and the risks associated with the processing, transfer, and storage of personal data or other sensitive information. Personal data and additional sensitive information, such as biometrics and medical records, must be sent securely across IoT networks. According to the European Union’s General Data Protection Regulation (GDPR) [7], other data that has been designated as personal data include unique device identifiers, IP addresses, unique identifiers of mobile providers, and wireless access points.

Several attack vectors are currently being investigated in the IoT environment, including but not limited to denial of service (DoS), distributed denial of service (DDoS), web attacks, infiltration, address resolution protocol (ARP) poisoning, and portscans, which can either occur at the perception, transport, or application layers of the network [8]. Due to the high negative impact of any successful attack on the enterprise network and organizational relevance, companies now rely on intelligent IDS models to automatically detect any intrusive attempts on their networks to reduce massive losses; hence, leading to more intensive research in the area of IDS for computer networks [9].

The effects of these attacks have recently resulted in massive data losses, especially those caused by the DDoS [8] which has newly generated severe damages to IoT systems [10]. DDoS attacks in their various forms can bring down an entire network by using up all of its resources by sending too many get requests that are more than the target system can handle. These attacks work mainly by taking advantage of multiple operating systems to break into them and use them as agents to send a lot of traffic to the target client. Other attacks have also shown high levels of negative impacts on computer networks and other computing devices [11]. IoT devices, fog devices, workstations, and more are some of the many vulnerable systems that can be compromised or exploited [10, 12].

In response to the threat that these attack vectors pose, many methods have been looked into to make sure that the IoT network is safe from all kinds of cyber threats. Approaches like machine learning (ML), deep learning (DL), intrusion detection systems (IDS), and intrusion prevention systems (IPS) that are based on artificial intelligence (AI) are some of the most searched-for topics in this field [13–15]. At the instance of detection of malicious network flows, IDS can take immediate action to deter the attack from spreading to the entire IoT network [16]. IDS are generally divided into two categories based on the detection mechanism used in their design: anomaly and misuse detection [11]. During the analysis of how network profile activities change over time, flows that could be harmful to the network can be found to be acting in strange ways. Even though they have a lot of false positives (FPs), anomaly detection IDS can find new and different types of attacks. On the contrary, misuse detection is able to distinguish between legitimate and dangerous attacks using previously established patterns; hence, it can accurately identify known attacks but has limitations with new and unknown attacks [11].

With the advent, availability and easy access to large-scale data, highly-performing hardware accelerators, state-of-the-art machine learning, and deep neural networks (DNNs), predictive modeling for problem-solving can now be performed at a large scale, especially in the IoT ecosystem, where large volumes of data are generated and transmitted along a resource-constrained channel [17]. The majority of the datasets used in IDS modeling tasks such as the NSL-KDD, KDD Cup’99, and UNSW-15 contain high dimensionality, which means they have a large number of features. This makes it harder to process these datasets. In order to solve the problem relating to data dimensionality, the most common approach has been the use of singular value decomposition-enabled methods such as principal component analysis (PCA) [18] and isometric feature mapping (Isomap) [19]. The main problems with using PCA and Isomap to reduce the number of features in a dataset are linear embedding and essential feature loss, even though they produce good results and keep the distances between data points to a moderate degree. Hence, they are not most suitable for dealing with the problems of the curse of dimensionality [19]. In [20, 21], the authors presented different methods of reducing the dimensionality and features of the CIC-IDS2017 dataset in an attempt to reduce the computational cost and improve the speed of model performance.

Furthermore, there is the challenge of explaining the relationship between the various features interacting in the dataset to produce the results from the model. In the cybersecurity atmosphere involving network intrusion detection and prevention, where the predictions of the model are of grave importance to the network administrator and the safety of connected devices, it is imperative that AI models be able to automatically provide explanations for their decisions. This is imperative because any misrepresentation or misinterpretation of the model’s performance can cause a devastating loss of the network, data, and other relevant information. Since it is important that ML model predictions continue to be as transparent and dependable as possible in an interpretable way, many model-specific and model-agnostic techniques, including both local and global interpretability, have been applied recently [22]. While the local explanations emphasize individual predictions, the global explanations provide detailed insights about the entire model’s behavior in the form of plots or more interpretable approximations such as decision boxes. Some of the commonly used methods to achieve model explainability include Permutation Feature Importance (PFI) [23], SHapley Additive exPlanation (SHAP) [24], Contextual Importance and Utility (CIU) [25, 26], and Local Interpretable Model-Agnostic Explanation (LIME) [27]. The explanations provided by these methods show high reliability in validating the performance of the model, which can be measured in terms of the accuracy, consistency, and stability of the unseen data [28].

Thus, there is a need for a tabular data-centric algorithm that provides both automatic feature selection and explainable results on the choice of the features used in the model development and the predictions of the model applied to IoT security. Considering the limitations of the existing deep learning-based model to provide interpretability to the model decision without third-party frameworks and large resource requirements, this research specifically addresses these issues categorically. Therefore, we propose an attentive transformer deep learning algorithm for IDS that implements automatic feature engineering and selection for detecting threats in an IoT ecosystem. We develop the IDS model using the TabNet [29] architecture. Different metrics, like accuracy, precision, recall, prediction time, and Matthew’s correlation coefficient, are used to judge how well the model works. We look at how well the model can be explained using the masking feature, which gives us the decision output. Specifically, the major contribution of the paper includes:

- The proposed IDS model introduces a unique approach to feature engineering and selection by incorporating an attention mechanism. This address the challenge of handling a large number of features, which can result in decreased model performance as a result of noisy and irrelevant features. The attention mechanism permits the model to learn and select which features most importantly contribute to the model overall behavior; leading to a more efficient and accurate IDS model to detect and prevent security breaches.
- Provides a solution to the challenge of interpretability of deep learning-based models that mostly depend on third-party frameworks to explain the behaviors of the model with respect to feature usage for predictions. By incorporating the ability of TabNet architecture to use a sparse feature selection process and learn interpretable decision rules, we provide an approach to automatic feature explanations which helps users understand the reasoning behind the predictions. Additionally, the explanations are used to improve the model by identifying the features that may require more attention in applications where the TabNet architecture is not implemented.
- Considering the resource constraints of the IoT system, the model is optimized using a lightweight optimization algorithm, the OPTUNA that is computationally efficient to determine the properties of the TabNet architecture most suitable for the IDS model. This ensures that the resulting model is suitable to be executed in resource-constrained environments and also for real-time applications for fast response.
- The model offers a balance between computational efficiency and accuracy, meeting the energy requirement of IoT devices. Adding to the lightweight and energy-efficient attributes of our proposed model, it requires less training and test time, making it ideal for real-time applications, as it can process data quickly and accurately, providing fast and reliable predictions.

In general, our proposed IDS model aims to prefer a solution to the limitations of existing deep learning-based models by providing a more inclusive and interpretable solution. The growth rate of IoT devices makes it critical to develop IDS models that not only show high accuracy but also provide insight into its decision rule through interpretability and explanations. This is believed to be of immense assistance to network analysts in analyzing zero-day attacks. Our proposed model emphasizes interpretability, which is critical for gaining trust and understanding the decision rule applied by the IDS model to network flows. By using modern optimization algorithms, we ensure that our proposed model provides competitive results in accuracy while requiring fewer resources for implementation, making it a generalized solution for network security in IoT environments.

## Related works

Advances in communication technology, as well as the availability of various sensing and computational devices, have resulted in rapid growth in the field of IoT devices and their application areas. Because of the widespread use of smart devices across various domains, IoT security has become a source of major concern in terms of protecting users’ confidentiality and privacy, as well as the hardware and network of IoT systems. Several authors, including [30–32] gave a detailed overview of the current trends in IoT security, asserting that the high rate of adoption of the technology has paved the way for more threats to continue to ravage the IoT system. Despite the fact that numerous research projects are underway with the primary goal of improving the level of security of smart devices, the domain remains largely unexplored. When using some of the most widely available security tools, such as encryption, authentication, access control, network protection, and application control, time wastage is usually encountered coupled with the inefficiency of the tools to provide a comprehensive check against the inherent vulnerabilities.

It is possible for hackers to break into open networks that provide access to certain intelligent utility services (such as smart grid systems, communications systems, and healthcare systems), posing serious hazards of information leakage, service disruption, and financial losses [1]. Such attacks that take down the availability of networks and devices and which have been reported amidst other forms of threats to be most harmful to the networks are the DDoS attacks. For instance, Mirai is an uncommon sort of botnet that triggers huge-scale distributed denial-of-service (DDoS) strikes by abusing IoT machines [33] such that they become unable to respond to user requests, thereby becoming exhausted and eventually shut down. The Persirai thingbot is one variant of Mirai code that continues to grow and infect Internet Protocol (IP) cameras [34].

In [35], authors propose an IDS model in IoT networks using deep learning algorithms specifically designed to detect DoS attacks in two main domains: passive and active threats using different algorithms, including CNN and MLP. The BoT-IoT dataset was used as a testbed to develop and evaluate the model’s performance, using accuracy, recall, and precision measurement as the performance evaluation metrics, with the CNN reaching an average accuracy of 91.27% and MLP achieving 79.01%. With the high accuracy, there remained an issue with understanding the basic features of the dataset that prevailed over the model’s decision. Hence, many ML and DL models remain in the black box domain where there is no thorough explanation of the decision of the model. The interpretations provided by some frameworks about the model decision can be identified as either local or global interpretations. A local interpretation provides insight into the model’s prediction based on each feature of the dataset. On the other hand, when the interpretation is made globally, the explanations result from the entire set of features in the dataset [36]. LIME is well known for its ability to provide local interpretability while SHAP is widely used for global interpretations.

In [37], Ensemble modeling was used to classify network intrusions. The research used the “Network Socket Layer—Knowledge Discovery in Databases” records (NSL-KDD). The authors converted the network flows into a malicious and non-malicious network packet to perform a binary classification task. The research used an ensemble model termed extreme gradient boosting (XGBoost) to identify records based on NSL-KDD input attributes. The XGBoost-based classification model was trained on 125973 and tested on 22544 records with 98.7% accuracy. Leveraging the data from the UNSW-NB15 dataset obtained from a publicly available data repository, authors in [38] performed a machine learning intrusion detection performance analysis and compared different machine learning techniques for detecting network intrusions, including SVM, Naïve Bayes, Decision Tree, and Random Forest. Based on their analysis of the UNSW-NB15 dataset, the authors opined that the SVM, Naïve Bayes, Decision Tree, and Random Forest achieved a detection accuracy of 92.28%, 74.19%, 95.82%, 97.49%, respectively. These results are black-boxed as it does not provide an insight into the different network flows or packets that defined the model’s prediction results.

Because of how complicated the IoT ecosystem is and how much data it generates, each piece of data has a huge impact on how the network flows. This has made it more important to understand how each piece of data helps predict whether the network profile is normal or not. Model explainability is an approach to solving this challenge. In [22], Deep learning algorithms for detecting network intrusion in IoT devices were proposed. Authors leveraged the explainable characteristics of some explainable AI (XAI) frameworks, such as LIME and SHAP to provide explanations for the model’s decisions. The problem with this method is that it needs a lot of computing power because SHAP values are hard to compute. On the NSL-KDD dataset, the proposed models achieved a performance of 82.4% and 96.4% in accuracy and precision, respectively, in a binary classification task. The authors in [36] proposed an approach for model interpretation specifically for security systems called LEMNA. LEMNA was tested on a malware dataset using a deep learning algorithm that takes an input data sample and produces a minimal set of interpretable characteristics that can be used to explain the classification of the input data sample. The central concept is to use a straightforward, interpretable model to approximatively represent a small region of the infinitely complicated decision boundary of deep learning. To better function with security applications (such as binary code analysis), the local interpretable model is optimized to (1) manage feature dependency and (2) handle nonlinear local limits, which improves explanation fidelity.

In both supervised and unsupervised language modeling problems, the ability of attention-based mechanisms to find larger subsets of features used for modeling has been used to improve the accuracy of the predictive model. One of the attention mechanisms is the self-attention network (SAN) [39, 40] which identifies important features primarily from tabular data. Meanwhile, this technique has been found to be more efficient with datasets that have a larger amount of feature space, showing very lower performance with datasets having fewer feature dimensions, therefore, implying that a limited amount of data can impact the relevant part of the feature. TabNet-IDS [29], is proposed, which chooses meaningful features in a dataset to reason from using a sequential attention mechanism in each step of the decision process during the model’s training. It provides a visualization of the feature’s importance and demonstrates how each feature contributes to the prediction made by the model. The mask attribute provides both local and global interpretability [41]. The summary of the related literature including the strength and weaknesses are shown in Table 1. Except for our proposed model, none of the works in the literature utilized optimization techniques to reduce the complexity of the model and improve performance.

**Table 1:** Summary of related literature and their features.

Authors	Implemented Algorithm	Xplainable model	Optimization
Susilo and Sari [35]	CNN/MLP	No	No
Dhaliwal et al [37]	XGBoost	No	No
Belouch et al [38]	ML (DT,RF,SVM)	No	No
Mane and Rao [22]	DL	No	No
Yang et al [42]	BiLSTM	Yes (LIME)	No
Singh et al [43]	GRU	No	No
Chen et al [44]	Tab-SRU	Yes (Self)	No
Yin et al [45]	RNN	No	No
Our study	TabNet-IDS	Yes (Self)	Yes

## Methodology

**Fig 1:**
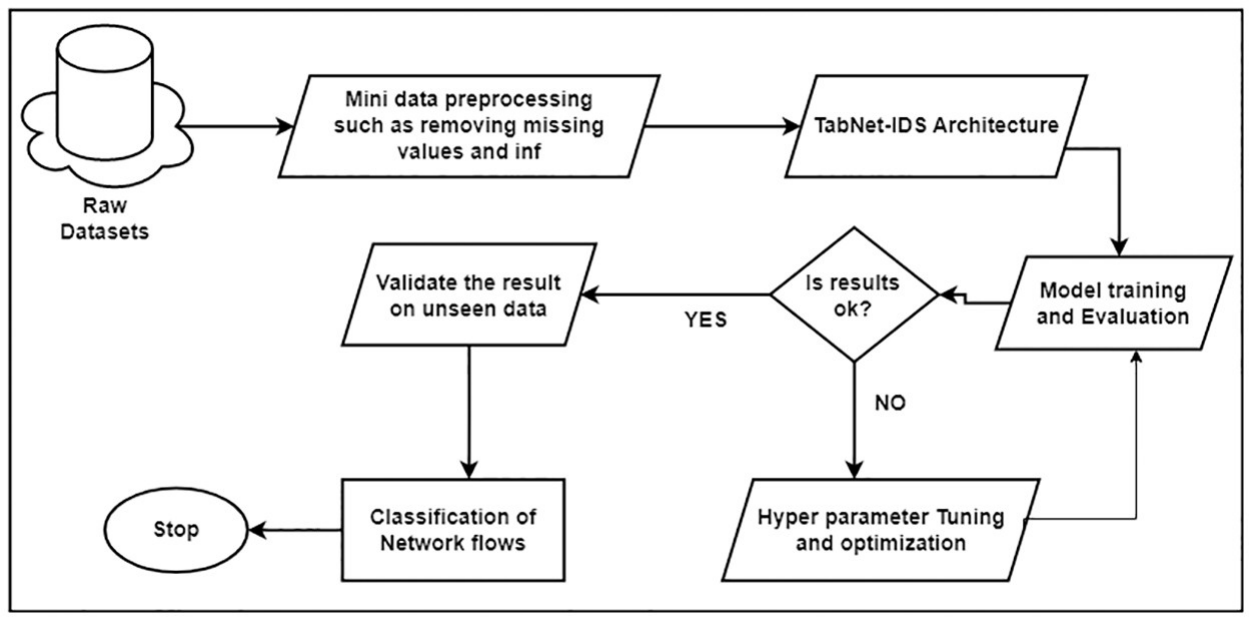
Flowchart for the design of the proposed TabNet-IDS model in this research.

This section discusses the datasets, and preprocessing approaches, the proposed algorithmic architecture, and the optimization techniques adopted to obtain suitable data for modeling for model development. The overall workflow used for this design is as shown in Fig 1.

### Dataset description

Three different IDS datasets, including CIC-IDS2017, CSE-CIC-IDS2018, and CIC-DDoS2019 are used to evaluate the performance of the proposed model. These datasets are general-purpose and relevant for developing predictive models for IDS in all computer networking scenarios. Considering the versed number of datasets available for training ML models, we have selected these three datasets considering that they are more recent and contain a larger number of network flows and attack profiles; hence provide an enabling ground for the model to learn from a vast feature set and data instances.

#### CIC-IDS2017

The CIC-IDS2017 is a general-purpose dataset that is widely used in the cybersecurity domain for modeling predictive IDS for IoT and computer networks. It is widely used among researchers because it was the first realistic yet comprehensive dataset proposed [46]. The dataset contains 83 features and 15 classes, collected using different machines over a period of seven days. A summary of the instances of the dataset and different classes with the percentage contribution is shown in Table 2. Just like other datasets, CIC-IDS2017 suffers from data imbalance, with a total of 83% of the entire network traffic belonging to normal traffic and only 17% being attacks. On analysis, we discovered that CIC-IDS2017 has 288602 instances with missing class labels and 2013 instances with missing information. These unwanted instances are dropped to form a new dataset that contains unique 2830540 instances. for the purpose of our experiment, a sample of the dataset is used after handling the imbalance as described in [11].

**Table 2:** Distribution of stream records in CICIDS2017 dataset.

Label Name	Value	Percentage (%)
Benign	2359289	83.3452
DoS Hulk	231073	8.1630
PortScan	158930	5.6144
DDoS	41835	1.4779
DoS GoldenEye	10293	0.3636
FTP-Patator	7938	0.2804
SSH-Patator	5897	0.2083
DoS slowloris	5796	0.2048
DoS Slowhttptest	5499	0.1943
Bot	1966	0.0695
Web Attack—Brute Force	1507	0.0532
Web Attack—XSS	652	0.0230
Infiltration	36	0.0013
Web Attack—SQL Injection	21	0.0007
Heartbleed	11	0.0004

#### CSE-CIC-IDS2018

This is a general-purpose dataset constructed using different synthetic communication networks and captured about 2.8 million labeled data (malicious flows) with 80 features, thereby increasing the complexity of the dataset significantly compared to those before it. In total, the dataset contains over 16 million network flows collected over 10 days from different devices and stored as a PCAP file. Because this dataset contains more network profiles, it tends to give more realistic flow behavior, and thus, IDS trained with this dataset tends to learn more on the available feature instances. The dataset contains 16 different classes and 80 features, with the highest number of instances representing the benign class, which mimics normal network communication over the IoT network. The remaining 15 classes represent different attack types to the tune of 18% of the entire dataset, hence showing a high rate of imbalance [11] as shown in Table 3.

**Table 3:** Distribution of stream records in CSE-CIC-IDS2018 dataset.

Label Name	Value	Percentage (%)
Benign	13484708	83.07001
DDoS attack-HOIC	686012	4.22605
DDoS attacks-LOIC-HTTP	576191	3.54952
DoS attacks-Hulk	461912	2.84552
Bot	286191	1.76303
FTP-BruteForce	193360	1.19116
SSH-Bruteforce	187589	1.15561
Infiltration	161934	0.99756
DoS attacks-SlowHTTPTest	139890	0.86177
DoS attacks-GoldenEye	41508	0.25570
DoS attacks-Slowloris	10990	0.06770
DDOS attack-LOIC-UDP	1730	0.01066
Brute Force -Web	611	0.00376
Brute Force -XSS	230	0.00142
SQL Injection	87	0.00054

#### CICDDoS2019

The CICDDoS2019 dataset is the most recent dataset that describes different DDoS attack profiles that are applicable to both IoT systems and computer networks. The dataset was collected from a public repository at the University of New Brunswick, Canada [8]. Various experiments involving classical ML algorithms and DL methods have already been performed using this dataset, but none specifically considers the problem of using a DL classifier specific to tabular datasets to analyze the dataset. Also, the problem of feature engineering has always been handled manually in previous works, unlike ours, which uses an attention mechanism to automatically select the salient features to reason from during the training and testing phases. Overall, the dataset contains about 50,063,112 records, comprising 56,863 benign traffic profiles and 50,006,249 malicious network flows during the training phase. In the testing phase, a total of 20,364,525 records were obtained, with benign flows of 56,965 and 20,307,560 malicious traffic records. The entire dataset contains 88 unique features extracted from the packet capture (Pcap) files using the CICFlowMeter [47], a network flow generator and analyzer. The first eight features of the dataset, including the source IP, destination IP, source port, destination port, flow ID, timestamp, and protocol, are the default parameters of the CICFlowMeterV3, while the remaining eighty features define the behavior of each of the flows. As noted in the work of [8] who originally generated the dataset, the training dataset contains DDoS attack vectors including, NTP, DNS, LDAP, MSSQL, NetBIOS, SNMP, SSDP, UDP, UDP-Lag, WebDDoS, SYN, and TFTP. Meanwhile, the testing dataset contains only seven (7) DDoS attack types, with the inclusion of a new attack profile that is absent from the training set. The attack vectors are PortScan, NetBIOS, LDAP, MSSQL, UDP, UDP-Lag, and SYN.

For a better understanding of the different attack profiles, they are grouped into two sets, including the reflection-based and exploitation-based attacks. In reflection-based attacks, the attackers use third-party products or components that they legitimately purchased to conceal their identities. The application layer protocol is often used to send both malicious and normal network packets to reflector servers [8]. The attackers set the source IP address of the attacking agent to be the same as the IP address of the victim or target system. This makes it look like the attacker is a real user. This attempt overwhelms the victim’s device and sends a lot of responses until the victim’s device can’t handle it anymore. This is further sub-grouped into TCP-based attacks, UDP-based attacks, and TCP/UDP-based attacks. The TCP-based attacks, like the MSSQL and SSDP profiles, can only be carried out with the TCP protocol. Attacks that use the UDP protocol are called UDP-based attacks, and they include the CharGen, NTP, and TFTP attack vectors. Also, some DDoS attacks can be carried out using either the TCP or the UDP protocols. These are DNS, LDAP, NetBIOS, and SNMP vectors.

**Fig 2:**
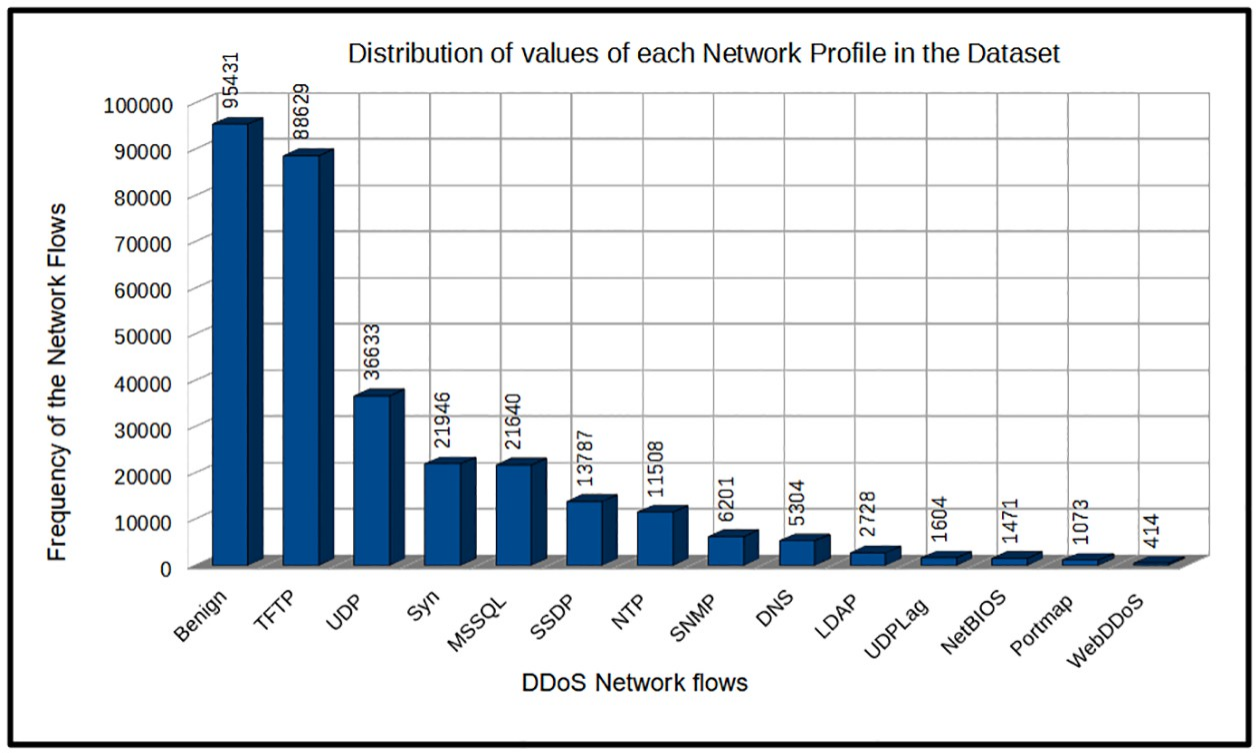
Distribution of the 10% of the CIC-DDoS2019 use for the training and testing of the model.

Even more, the same method of hiding identity is used in exploitation-based DDoS attacks. However, the major difference lies in the primary focus of the attack points. In this kind of DDoS attack, the attackers try to take advantage of certain protocols, such as the network, application, and transport layers of the Open Systems Interconnection (OSI) model. Cyberattackers send packets to the servers bearing the source IP addresses configured to the victim’s IP address to overburden the victim with response packets. This DDoS attack type is also grouped into the TCP-based attacks that comprise SYN Flood and the UDP-based attacks that include UDP Flood and UDP-Lag. Due to the large volume of the dataset, we used 10% for our research as shown in Fig 2.

### Data preparation

In most cases, network flows arrive in a very jumbled condition that has to be cleaned up before they can be used. It is still the most important step in machine learning since the quality of the dataset affects how well the model works. In the proposed architecture, the TabNet-IDS, the major data preparation process is automatically handled within the internal mechanism of the algorithm. Therefore, only basic data cleaning, like getting rid of duplicates, deleting and filling in missing values, and encoding categorical characteristics have been handled in this phase. The training of the model can be improved leading to an easy learning process if the labels are encoded into their numerical equivalent. By scaling the data, we were able to collect data points that are very close to one another, which helped remove any bias from the final forecast. In addition to this, it helps keep the data’s integrity intact over time. Another important part of the process of cleaning up the data is figuring out how to deal with the problem of imbalance which is commonly observed in any real-world dataset. Several different methods, like the Synthetic Oversampling Technique (SMOTE) and its variants including, the Adaptive Synthetic (ADASYN) algorithm, SMOTEN, SMOTEN, etc. have been suggested as possible solutions to this problem. However, we take care of problems caused by imbalance automatically within the proposed TabNet-IDS model using the built-in augmentation fit function argument called classificationSMOTE. By setting this argument to true, the model automatically takes care of any issues caused by an imbalance within the training data, ensuring that it is balanced between different classes.

### Overview of proposed model and implementation

#### Sequential attention model

Tabular data is one of the most readily available types of datasets used for ML tasks. They are arranged in rows and columns and are most often saved as a comma-separated value (CSV) file. DNN models have shown good results on image, audio, and text data utilizing canonical architectures that permit the encoding and communication of the data in different formats [1]. Despite the fact that tabular data contains multiple data types that can easily function well with the canonical architecture, tabular-data-specific deep learning models have yet to be proposed for IDS.

Basically, IDS models developed with tree-based algorithms such as decision trees, random forests, XGBoost, LightGBM, Extra Tree classifiers, and deep learning models, including Deep Neural Networks (DNN), Recurrent Neural Networks (RNN), Convolutional Neural Networks (CNN), [11] etc require that the input tabular data be first feature engineered before it is fed into the algorithm for training. With TabNet, which provides a sequential attention mechanism in the model, the following benefits are inherent [29]: (i) automated preprocessing of the raw dataset, which is then trained using gradient descent-based optimization, thereby, allowing a more flexible data integration. (ii) A sequential attention-enabled feature selection technique carried out in a step-wise and instant-wise fashion. The instant-wise feature selection helps to reduce model complexity train time in comparison with other tree-based classifiers and this is achieved by using a single deep learning architecture, and (iii) a mechanism that encourages the design of lightweight models for IoT systems.

**Fig 3:**
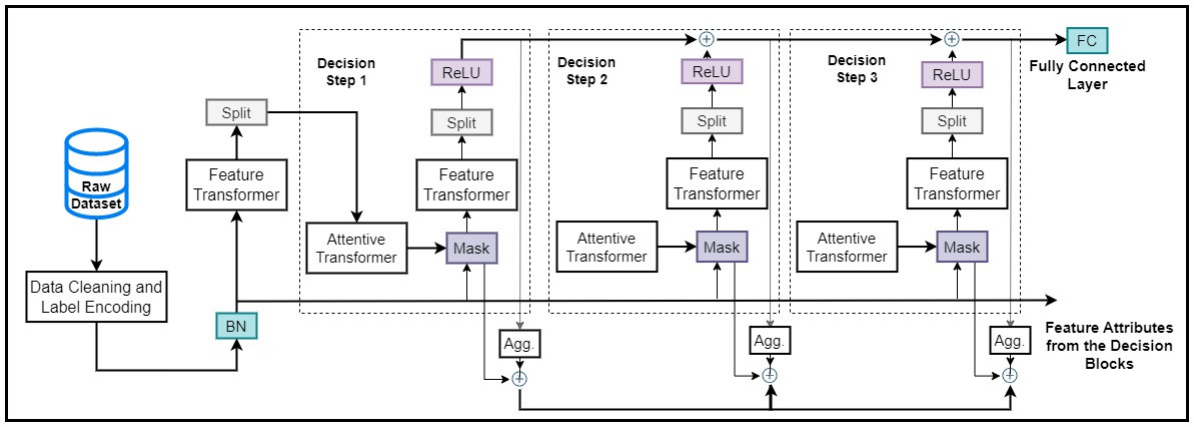
The TabNet encoder is made up of three parts: A feature transformer, an attentive transformer, and feature masking. The information to be used by the attentive transformer and the next feature transformer is separated with a split block. For each phase, the feature selection mask gives interpretable information regarding the model’s functionality, and the masks can be combined to get global feature significance attributes.

**Fig 4:**
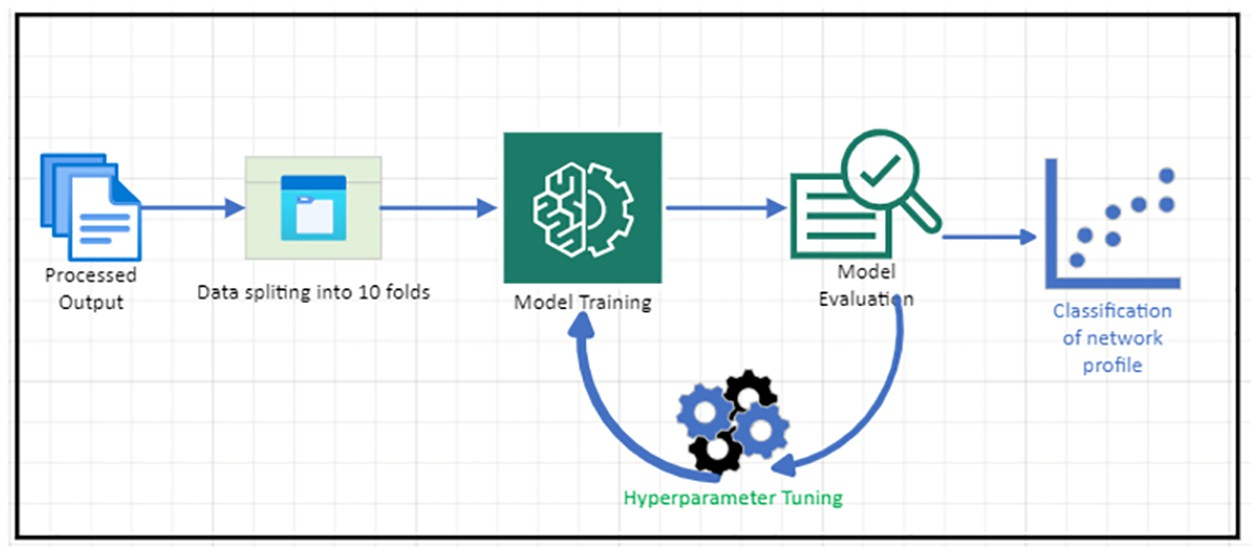
Model training, optimization, and evaluation.

The proposed IDS is implemented with the TabNet architecture, which is an algorithm specifically developed to train models on tabular data. Normally, individual feature selection is done by ML engineers when handling problems associated with DTs and DNNs algorithms. This is important for getting decision boundaries in the hyperplane, which can be thought of as a generalized linear combination of features with coefficients that say how much of each feature to use. TabNet uses this kind of functionality paradigm and also takes care of the problems of selecting features, processing features, and understanding them. Basically, the TabNet architecture exists in four different segments: the encoder, decoder, feature transformer, and attentive transformer modules, as shown in Figs 3 and 4.

As inputs, we give the architecture raw numerical features that have been slightly cleaned up and embeddings that can be trained for the mapped categorical features. The global feature normalization is not implemented, but we use batch normalization (BN). We implemented batch normalization as it generally improves the performance of deep learning models by normalizing the input layers and adjusting the activation of preceding layers prior to exchanging the information to subsequent layers. Specifically, in our study, batch normalization is used in each of the decision steps to improve the quality of the sparse feature selection process. On the output of the feature transformer, we also used batch normalization to stabilize the distribution of the transformed features, thereby reducing the impact of the initialization of the network data and encouraging the network to learn better representations of the input data. During the training phase, batch normalization computes the mean and variance of the activation for each of the mini-batch of the data and then normalizes the activation. This is done by subtracting the mean and dividing it by the standard deviation. This is to ensure that the mean and variance of the activation remain consistent through different batches, thereby ensuring that overfitting is reduced and improving the overall generalization of the model.

Given dimensions D and batch size B, the features can be expressed as **f** ∈ ℜ^*B*×*D*^. We pass these same dimensional features to each of the decision steps using TabNet encoding that is based on sequential multi-step processing with *N*_steps_ decision steps. The *i*^*th*^ step uses the processed network traffic from the (*i* − 1)^*th*^ step to decide which features to use and to output the processed feature representation, which is then aggregated into the overall decision. The output of Fig 3 is used as an input to the second phase shown in Fig 4 for the training process. First, the data is split into 10 folds, and in each of the training phases, 9 folds are used, while one is used for validation. The trained IDS model’s accuracy is checked, and then, depending on the score, the model is hyper-parameter tuned and a final evaluation is performed.

A learning mask given by Eq 1 is used to perform a soft selection of the most salient features of the dataset while maintaining the learning capacity of the decision step, resulting in a model that is more parameter efficient. The mask is used to obtain the local and global interpretations of the model performance.

$$\begin{array}{c} M\left[i\right]\in {ℜ}^{B\times D} \end{array}$$ (1)

where *M*[*i*] is the mask of the *i*_*t*_*h* feature of the dataset considered most important at that instant in the decision block.

The masking process uses a multiplicative gating mechanism, which zeros out all the feature values that are not selected by the mask. This helps to create a sparsified feature map engineered to reduce the total number of features that are used for the final prediction and improve interpretability. The attention mechanism uses the function given in Eq 2 to compute the mask *M*[*i*].

$$\begin{array}{c} a\left[i-1\right]:M\left[i\right]=sparsemax\left(P\left[i-1\right]\right).h_{i}\left(a\left[i-1\right]\right)). \end{array}$$ (2)

where *h*_*i*_ is an FC and BN layer, *P*[*i* − 1] is the prior information and the sparsemax function [48] is used to give sparsity because it can map the Euclidean projection to the probabilistic simplex. Hence, it provides sparse features for explainability. The prior scale denoted as *P*[*i*] is us to track all the previous knowledge gained on each of the features during the learning process providing robustness to the model since it can not use a previously used feature. Mathematically, the expression for this function is as given in Eq 3.

$$\begin{array}{c} P\left[i\right]=\prod_{j=1}^{i}\left(\gamma -M\left[j\right]\right) \end{array}$$ (3)

where *γ* denotes a relaxation parameter that determines the flexibility of using each of the features in the various decision step of the algorithm. Where *γ* = 1, a selected feature is restricted to being used only at one decision step, but when the value of *γ* increases, a particular feature can be used over multiple decision steps. To encourage more robust feature selection, we calculate the sparse loss, which is a regulation technique that penalizes the model for selecting too many features at a given step, forcing the model to pay more attention to the most informative features. The equation for calculating the sparse loss is given by Eq 4. Essentially, the equation computes the negative logarithm of the mask entries that relate to the unselected features implying where *M*_*b*,*j*_[*i*] = 0

$$\begin{array}{c} L_{\text{sparse}\;}=\sum_{i=1}^{N_{\text{steps}\;}}\sum_{b=1}^{B}\sum_{j=1}^{D}\frac{-M_{b,j}\left[i\right]}{N_{\text{steps}\;}\cdot B}\;\text{log}\;(M_{b,j}\left[i\right]+\epsilon ) \end{array}$$ (4)

where *ϵ* is a small constant added to the mask entries to avoid taking the logarithm of zero. The selected features are preprocessed so that they can be used in the model training. This is achieved with the feature transformer block, which consists of a fully connected layer (FC) followed by batch normalization (BN) and a gated-linear unit (GLU). The output of the processed feature is split into two parts: one part is used for the decision step, and the other part contains information for the subsequent step, as determined by their interrelationship. This is shown in Eq 5

$$\begin{array}{c} \left[d\left[i\right],a\left[i\right]\right]=f_{i}\left(M\left[i\right].f\right) \end{array}$$ (5)

where $d\left[i\right]\in {ℜ}^{B\times N_{d}}$ and $a\left[i\right]\in {ℜ}^{B\times N_{a}}$ [29]. We improve the model’s parameter efficiency and its ability to learn by using a shared layer to keep features from being used more than once in different decision steps and layers that depend on those steps. We improve the training of a TabNet by using large batch sizes with the BN as well as the ghost BN (a virtual batch normalization) form by using a virtual batch size *B*_*v*_ and momentum *m*_*B*_ and aggregating the entire decision embedding using Eq 6

$$\begin{array}{c} d_{out}=\sum_{i=1}^{N_{\text{steps}\;}}\;\text{ReLU}\left(d\left[i\right]\right) \end{array}$$ (6)

where ReLU is the activation function. The implementation algorithm is shown in Algorithm 1.

**Algorithm 1** TabNet Algorithm

**Result**: Trained TabNet model

Initialize input features $X\in R^{n\times d}$;

 Initialize target labels $y\in R^{n}$;

 Initialize hyperparameters **h**;

 Initialize empty list of decision steps *L* = [ ];

 Initialize empty list of feature masks *M* = [ ];

 **for** *t* = 1, 2, …, *T* **do**

  Feature masking

  **M**_*t*_ ← createMask(**X**, **h**);

  *M*.append(**M**_*t*_);

  Feature transformation

  **X**_*t*_ ← **X** ⊙ **M**_*t*_

  **A**_*t*_, **E**_*t*_ ← encode(**X**_*t*_, **h**)

  Decision step

  *d*_*t*_ ← decision(**A**_*t*_)

  *L*.append(*d*_*t*_)

  Update feature importance

  **X** ← **X** − **E**_*t*_

  **y**_*t*_ ← predict(**A**_*t*_)

  **X** ← updateFeatures(**X**, **y**_*t*_, **h**)

**end**

Train final layer on **A**_*T*_;

 **return** *Trained TabNet model*

In this algorithm, *n* represents the number of data instances and *d* represents the number of input features. **h** represents the hyperparameters of the model, including the number of decision steps *T*. createMask() is a function that creates a feature mask matrix for each decision step, which is applied element-wise to the input features to obtain masked features **X**_*t*_. encode() is a function that applies the encoder layers to the masked features to obtain feature attention masks **A**_*t*_ and transformed features **E**_*t*_. decision() is a function that computes the decision for each decision step based on the feature attention masks **A**_*t*_. updateFeatures() is a function that updates the importance of each feature based on its contribution to the predictions. Finally, the trained model is obtained by training a final layer on the last feature attention mask **A**_*T*_.

### Hyperparameter tuning with optuna

**Fig 5:**
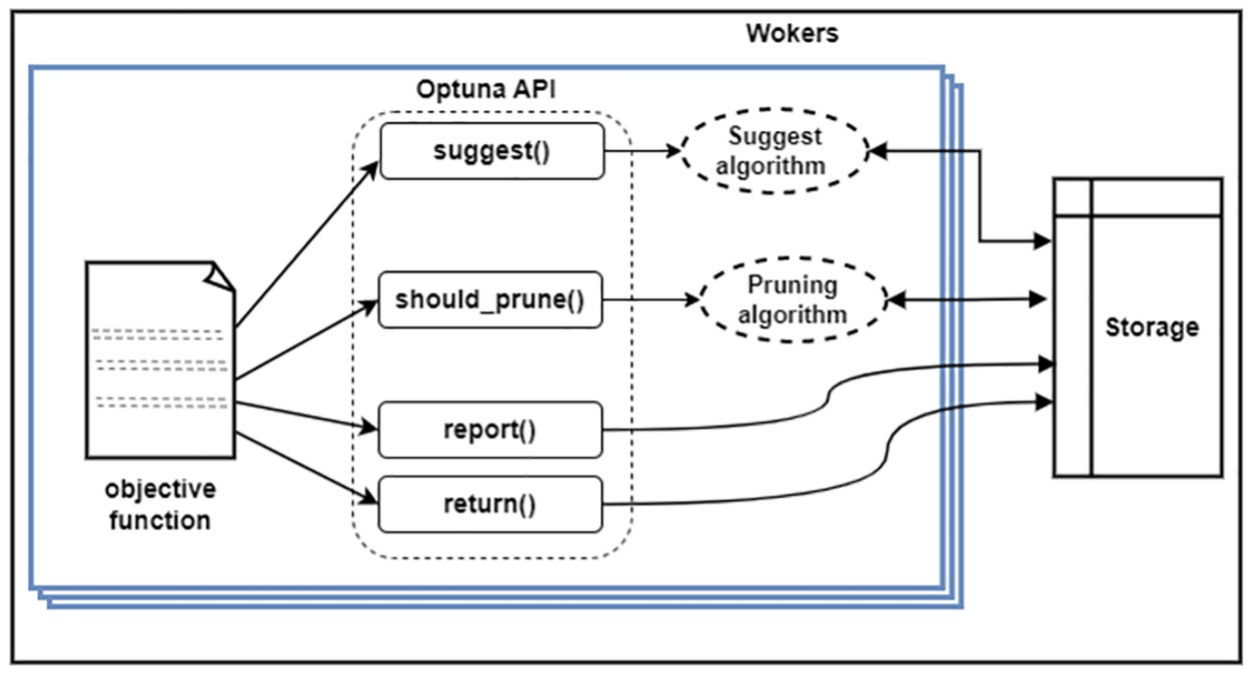
Optuna’s general system design. In the search study, each worker is responsible for executing one instance of the objective function. The objective function uses Optuna APIs to run its trial. The objective function accesses the shared storage to obtain information about past studies when necessary. Each worker operates independently, running the objective function and sharing the progress of the current study through the shared storage.

One of the most important parts of the TabNet algorithm is that it can explain why certain features were chosen at each step. We implement the design using the PyTorch framework with the Pytorch_tabnet module. PyTorch TabNet has a plethora of arguments that aid performance definition. In Table 4, we present the parameters of the algorithm and their default values. We use a search algorithm called Optuna to perform hyperparameter tuning. Optuna helps us recursively search a given space to find the best parameters that give the most reliable performance. Optuna [49] is an optimization algorithm that: (i) offers dynamic construction of the search space, hence, a define-by-run programming interface; (ii) is applicable to both lightweight and heavy-weight computational tasks due to its easy-to-setup architecture and low computational cost; and (iii) permits reduced time complexity with the use of pruning and efficient sampling paradigm [49]. The internal architecture of the optuna API is shown in Fig 5 adapted from [49].

**Table 4:** Description of TabNet hyper-parameters and optimized space.

TabNet Model Parameters	Description	Search Space	Search Result
*n*_*d*	The width of the decision prediction, default value = 8	[8,64]	60
*n*_*a*	Attention embedding for each mask, default value = 8	[8, 64]	60
*n*_*steps*	Denotes the number of steps in the architecture	[3, 10]	3
*n*_*independent*	Defines the number of Gated Linear Units layers in each of the steps	[1, 5]	2
*gamma*	Coefficient for feature re-usage in the mask. A value close to 1 makes mask selection least correlated between layers	[1.0, 2.0]	1.5
*n*_*shared*	The number of shared Gated Linear Units at each step. Default = 2	[1, 5]	3
*epsilon*	This value is not tuned	1e-15	-
*momentum*	momentum used for batch normalization, default = 0.02	[0.01, 0.4]	0.02
*lambda*_*sparse*	Adds extra sparsity loss coefficient to the model. A higher lambda sparse coefficient yields a more sparsed model in feature selection	[1e-3, 1e-5]	1e-3
*optimizer*_*fn*	PyTorch optimization function to reduce complexity and achieve minima, default = torch.optim.Adam	-	-
*scheduler*_*fn*	used to change the learning rate during training	-	-
*scheduler*_*params*	A dictionary of parameters to apply to the scheduler function	[“gamma”: 0.95, “*step*_*size*”: 10]	[“gamma”: 0.95, “*step*_*size*”: 10]
*mask*_*type*	defines the masking function to use for the feature selection, default = sparsemax	[“sparsemax”, “entmax”]	entmax
*Patience*	defines the number of consecutive epochs under which the early stopping is called if there is no improvement in the evaluation metrics	[15, 30]	20

**Fig 6:**
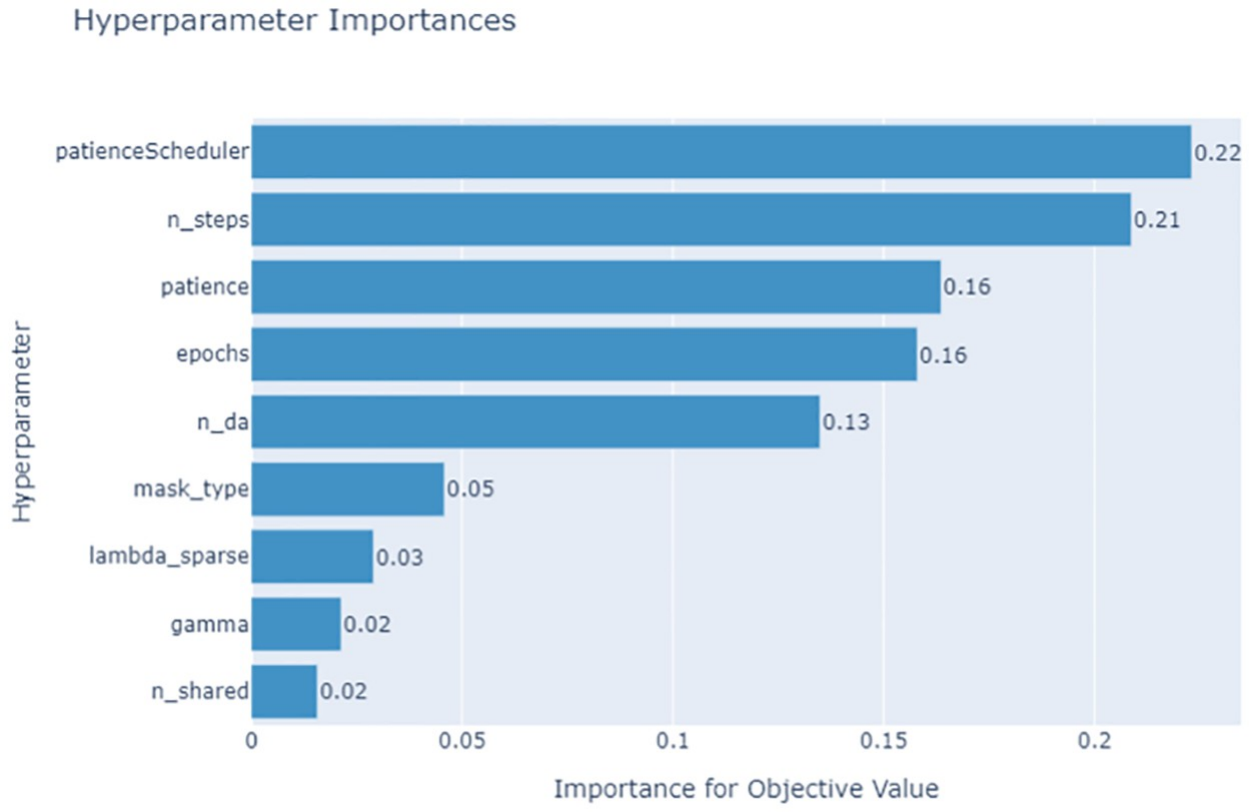
Hyperparameter importance of the model parameters obtained during the optimization process using optuna. *n*_*da*_ is used to represent the values for *n*_*d*_ and *n*_*a*_ since *n*_*d*_ = *n*_*a*_ is ideal for better performance.

We used three different steps to implement the optimization algorithm. First, we set the parameters for the search. Then, we started the objective function that would be used during the optimization. Finally, we called the study function, which does the search and records the observations made. We use different graph networks to show the search history, which helps us understand how well the search algorithm works. The parameter importance obtained during the optimization process is shown in Fig 6. The Patience Scheduler which is an instance of the *schedulerfn* and defines the patience level for each of the optimization trails tends to be the most influencing parameter for the optimization process. This, however, is dependent on the dataset used. The number of steps, patience level, and epochs as well are contributing features that enhance the model accuracy. The general algorithm for implementing the tuning is shown in Algorithm 2.

**Algorithm 2** Optuna for TabNet-IDS Tuning

**Input**: Train and validation data, number of trials *n*, range of hyperparameters to be tuned

**Output**: Optimal hyperparameters

objective_function(trial)

Define the objective function to optimize

**Input**: Hyperparameters *hp* sampled from trial

**Output**: Validation accuracy of the model

*model* ← TabNetModel(…, **hp)

*model*.compile(loss=”, optimizer=‘adam’, metrics=[‘accuracy’])

*model*.fit((train data), epochs = hp[‘epochs’], batch_size = hp[‘batch_size’])

*val*_*loss*, *val*_*acc* ← *model*.evaluate(val_data, val_labels)

**return** val_acc

Initialize the Optuna study

*study* ← optuna.create_study(direction=‘maximize’) Run the trials

**for** *i* ← 1 to *n* **do**

 *trial* ← study.ask() *hp* ← […]

 **for** *param* ← trial.params **do**

  *hp*[*param*] ← trial.params[param]

 *val*_*acc* ← objective_function(trial)*study*.*tell*(*trial*, *val*_*acc*)

Print the best hyperparameters found by Optuna *best*_*params* ← study.best_params

**print**(‘Best Hyperparameters:’, best_params)

In this algorithm, the function *objective*_*function* defines the objective to optimize, which is the validation accuracy of the TabNet model. The hyperparameters to be tuned are defined by the *hp* dictionary, which is passed as arguments to the TabNetModel function. The hyperparameters to be tuned can be specified in the range of hyperparameters to be tuned input of the algorithm as the search space *S*. The *optuna*.*create*_*study* function initializes the Optuna study and the *study*.*ask*() function samples a set of hyperparameters for each trial. The *study*.*tell* function updates the Optuna study with the validation accuracy of the model for each trial and the *study*.*best*_*params* function returns the best hyperparameters found by Optuna.

## Results and discussion

The proposed architecture was implemented on three different datasets, including the CIC-IDS2017, CSE-CICIDS2018, and CIC-DDoS2019. A cross-validation of 10 folds was used for each training process, and the accuracy, precision, recall, f score, and Matthew’s correlation coefficient (MCC) are measured for each model and in each fold. With a batch size of 1024, Adam was used as an optimization function to train the models. We used a Ghost Batch Normalization (GBN) of 128 that served as a virtual_batch_size for mini-batching the training samples, which enhanced the training time for the algorithm. The results obtained for the three datasets are shown in Table 5, indicating the accuracy, precision, recall, f1-measure, MCC, and test time in both the training and testing phases.

**Table 5:** Performance of the TabNet model on the selected datasets according to the number of folds.

Folds	Dataset	Accuracy	Precision	Recall	F-Score	MCC	Test time (Sec)
1	CIC-IDS2017	0.9725	0.9732	0.9723	0.9719	0.9719	3.1900
	CSE-CICIDS2018	0.9563	0.9573	0.9563	0.9561	0.9561	2.0500
	CIC-DDoS2019	0.9840	0.9830	0.9820	0.9844	0.9780	1.0600
2	CIC-IDS2017	0.9733	0.9738	0.9733	0.9727	0.9727	4.1400
	CSE-CICIDS2018	0.9525	0.9549	0.9525	0.9521	0.9521	2.1100
	CIC-DDoS2019	0.9855	0.9840	0.9830	0.9844	0.9880	1.0910
3	CIC-IDS2017	0.9691	0.9696	0.9691	0.9685	0.9685	4.1600
	CSE-CICIDS2018	0.9555	0.9577	0.9555	0.9553	0.9553	1.8900
	CIC-DDoS2019	0.9845	0.9835	0.9828	0.9848	0.9788	1.0470
4	CIC-IDS2017	0.9691	0.9699	0.9691	0.9685	0.9685	4.1200
	CSE-CICIDS2018	0.9552	0.9557	0.9552	0.9551	0.9551	2.0000
	CIC-DDoS2019	0.9855	0.9855	0.9840	0.9849	0.9783	1.0100
5	CIC-IDS2017	0.9623	0.9638	0.9623	0.9613	0.9613	3.5300
	CSE-CICIDS2018	0.9569	0.9579	0.9569	0.9567	0.9567	1.8000
	CIC-DDoS2019	0.9860	0.9836	0.9827	0.9848	0.9780	1.0550
6	CIC-IDS2017	0.9724	0.9728	0.9724	0.9718	0.9718	3.4330
	CSE-CICIDS2018	0.9557	0.9575	0.9557	0.9556	0.9556	2.0200
	CIC-DDoS2019	0.9840	0.9837	0.9820	0.9844	0.9787	1.0910
7	CIC-IDS2017	0.9737	0.9743	0.9734	0.9731	0.9731	4.1700
	CSE-CICIDS2018	0.9567	0.9577	0.9567	0.9556	0.9557	2.2000
	CIC-DDoS2019	0.9840	0.9830	0.9820	0.9844	0.9780	1.0880
8	CIC-IDS2017	0.9666	0.9674	0.9666	0.9660	0.9660	3.5400
	CSE-CICIDS2018	0.9566	0.9554	0.9566	0.9564	0.9564	2.1100
	CIC-DDoS2019	0.9844	0.9834	0.9822	0.9844	0.9780	2.0880
9	CIC-IDS2017	0.9707	0.9716	0.9707	0.9701	0.9701	4.1600
	CSE-CICIDS2018	0.9556	0.9569	0.9566	0.9562	0.9562	2.1100
	CIC-DDoS2019	0.9845	0.9835	0.9820	0.9844	0.9780	1.0400
10	CIC-IDS2017	0.9732	0.9738	0.9732	0.9727	0.9727	4.2000
	CSE-CICIDS2018	0.9568	0.9580	0.9567	0.9555	0.9555	2.1200
	CIC-DDoS2019	0.9840	0.9835	0.9825	0.9844	0.9780	1.1070

**Fig 7:**
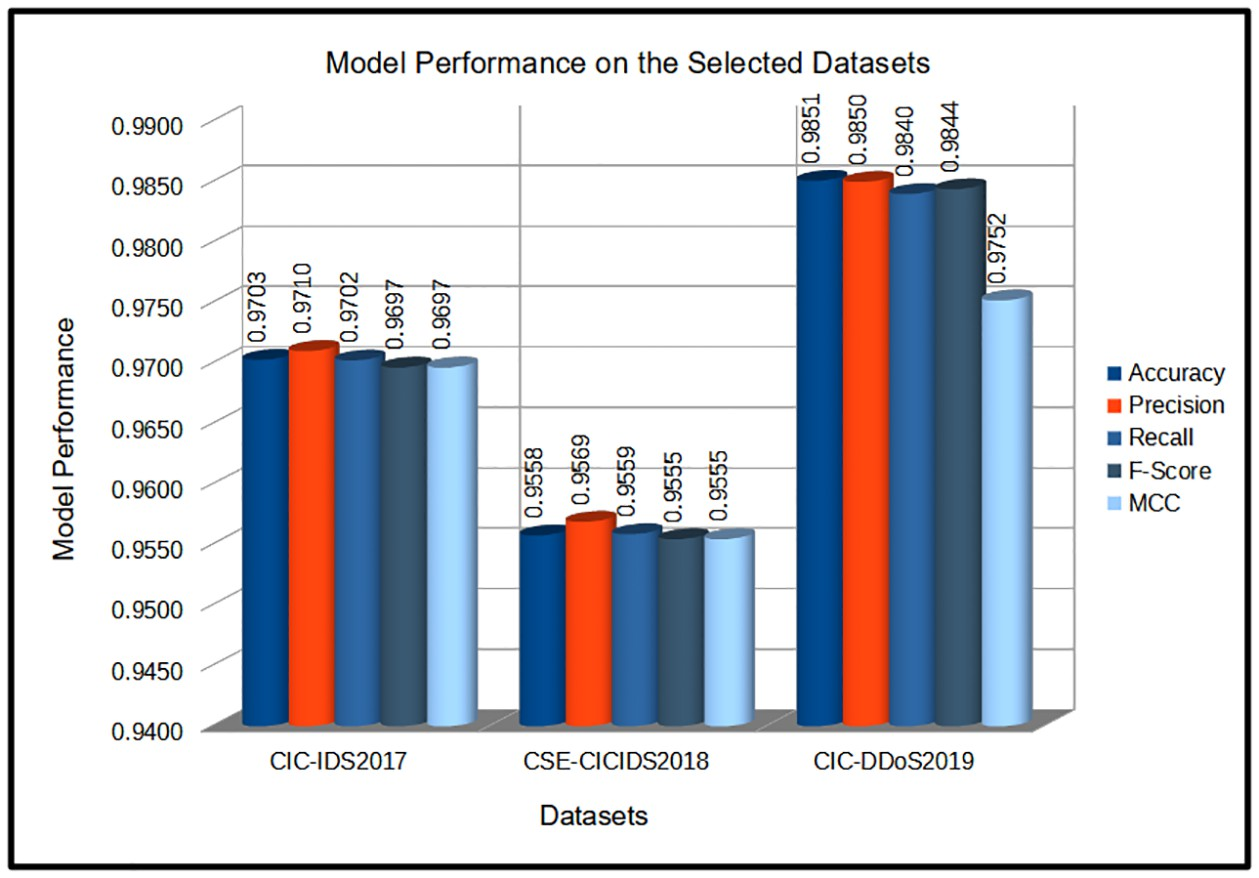
TabNet-IDS performance on the three datasets.

According to Table 5, the proposed TabNet-IDS model was evaluated using the three data sets which achieved different performance levels considering the evaluation metrics across the selected datasets. On the CIC-IDS2017 dataset, the proposed model achieved an average accuracy of 97.03% with a standard deviation of 0.36% (97.03%±0.36%), an average recall of 91.10% with a standard deviation of 0.33% (97.10%±0.33%), an average precision of 97.02% along with 0.35% standard deviation (97.02%±0.35%), F1-measure of 96.97% with standard deviation of 0.37% (96.97%±0.37%), and MCC score of 96.97% and standard deviation of 0.37% (96.97%±0.37%) The model’s testing time on this dataset is 3.86secs. Similarly, on the CSE-CIC-2018 dataset, the model shows an average accuracy, recall, precision, F-measure, MCC score of 95.58%±0.13%, 95.69%±0.11%, 95.59%±0.13%, 95.55%±0.12%, 95.55%±0.12% respectively. In each of the metrics, the corresponding standard deviation is 0.13%, 0.11%, 0.13%, 0.12% and 0.12% accordingly. The average test time of the model on this dataset is 2.04Secs. In the same way, on the CIC-DDos2019 dataset, similar performances are obtained for the accuracy, recall, precision, F-measure, and MCC score reaching 98.51%±0.12%, 98.50%±0.14%, 98.40%±0.21%, 98.44%±0.32%, 97.52%±0.33% respectively. The average test time on this dataset is 1.16 secs. The general model evaluation performance is shown in Fig 7. Consequently, the results indicate that the proposed model performs well on all three datasets, with the highest performance achieved on the CIC-IDS2019 dataset. The testing times for all three datasets were reasonable, indicating that the proposed model can efficiently process and classify network traffic data. However, it is important to note that the performance may vary depending on the specific characteristics of the dataset, and further evaluation of additional datasets may be necessary to validate the model’s generalizability.

Upon analyzing the performance of the Tabnet-IDS model on the selected datasets, we observed consistent results across the various evaluation metrics. This remarkable feature indicates the reliability and efficiency of the model’s predictions. More importantly, it is notable to state that the prediction time is significantly low, empowering the model to detect high-profile network flows accurately within a short period. As a result, the model can provide assistance in quick response to attacks on IoT devices and deter further malicious attacks.

**Fig 8:**
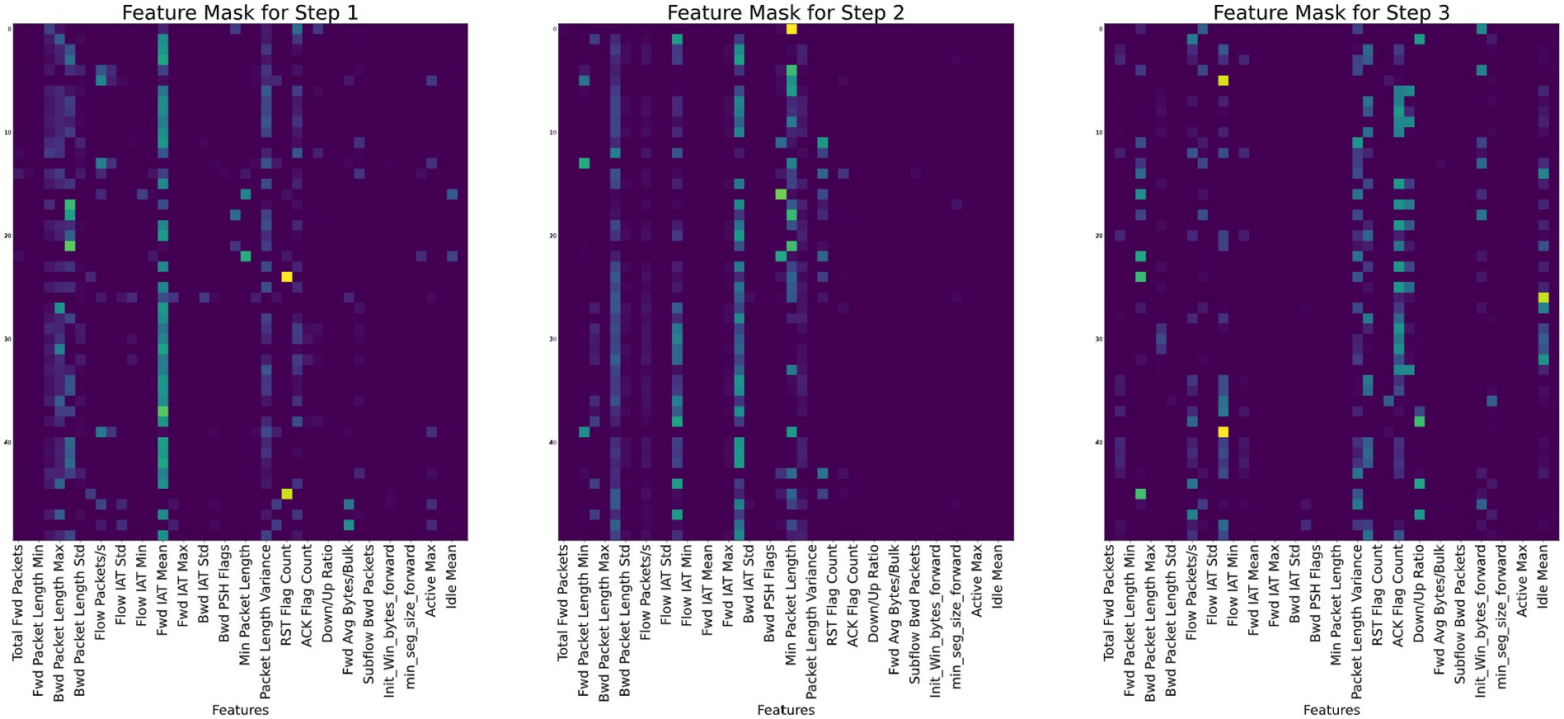
Feature masking output for the three decision steps of the TabNet model on the CIC-IDS2017 dataset.

**Fig 9:**
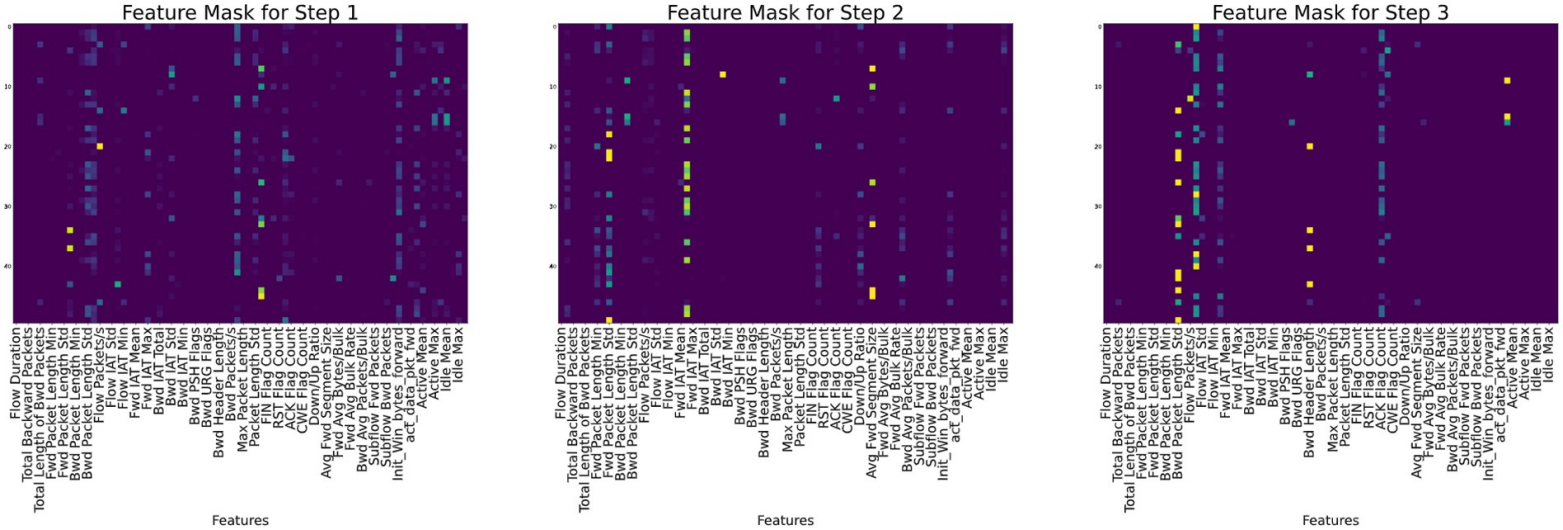
Global explanation for the selected features used in the CIC-IDS2018 dataset.

**Fig 10:**
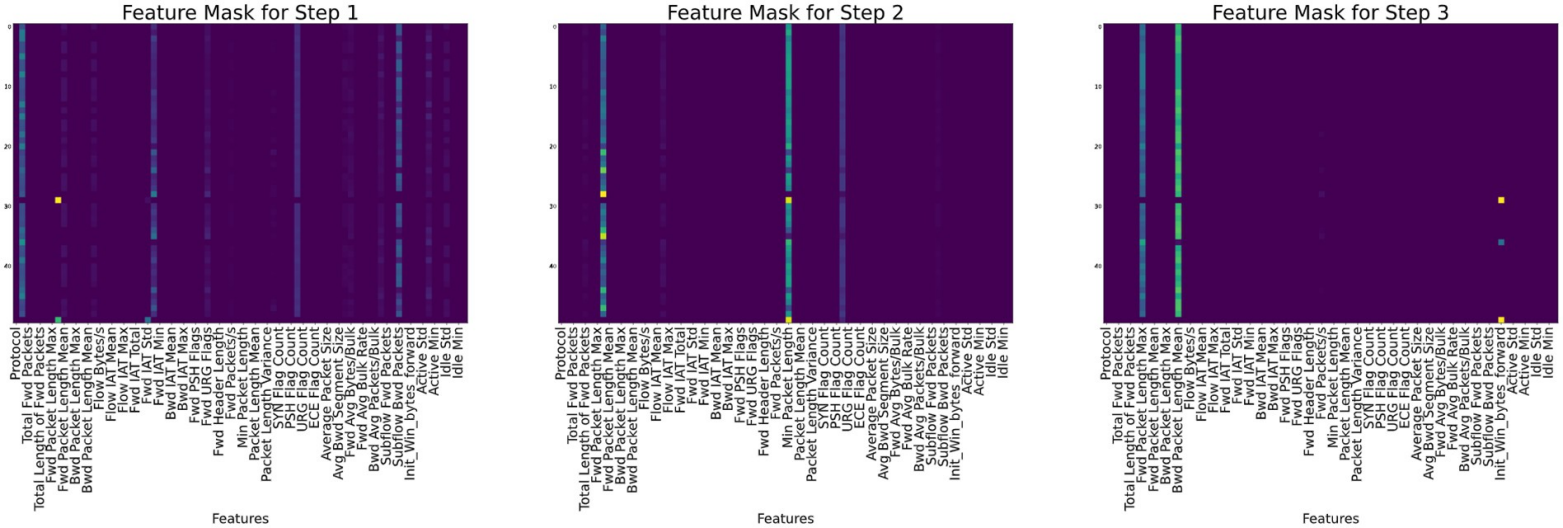
“Global explanation for the selected features used in the CIC-IDS2019 dataset”. Masks for each step show that the Fwd Packet Length Min, Fwd Packet Length Mean, Total Fwd Packets, Min Packets Length, Bwd Packet Length Mean, Fwd Packet Length Max, and Fwd Packet Length Std are mostly used for the decision in the Masks.

In terms of explainability, the various features [46] that were selected during each step by the mask are shown for each of the datasets in Figs 8–10 for CIC-IDS2017, CSE-CICIDS2018, and CIC-DDoS2019 respectively.

In each of the masks, different sets of feature values are selected from the dataset based on importance and used to train the model. The dark colors on the masks show the most essential parts, while the light colors show the less critical parts. In Fig 8 for example, features like Fwd IAT Mean, Bwd Packet Length Mean, Fwd Packet Length Std, and Bwd Packet Length Max, tend to show more prominence among all the features in deciding the model’s performance according to mask 1, while in mask 3, features like Flow Duration, Fwd IAT Mean, Flow IAT Min, Packet Length Mean, Down/Up Ratio, and Min Packet Length informed the decision of the model in that step. Similarly, in Fig 9, considering Mask 1, the instant-wise feature selection and reasoning almost utilized the entire features with Protocol, Pkt Len Mean, Flow IAT Std, Fwd IAT Std, Fws Seg Size Mean showing preeminence over all other features. The Flow IAT Std, Fwd Pkts/s, Pkt Len Std, and Ack Flag Cnt are seen to be more influential among the selected features in Mask. Each mask represents the shared decision step shown in Fig 3.

Each feature’s relative weight in the dataset is represented by its color in the feature column. Nevertheless, the relevance of characteristics may vary from sample to sample, as shown by the color of the feature column. Different samples prioritize various features, as seen by the global feature selection shown by the aggregate feature importance mask. Instead of really employing a decision tree, the TabNet approach uses a deep neural network to imitate one. This method is useful since it prevents rows with identical characteristics from always being shown.

In general, the proposed TabNet improves the detection and classification performance of the IDS model by leveraging its inherent features of adaptive feature selection, decision steps, sparse feature representation, gradient-based learning, and the ability to handle high-dimensional data. IDS model is able t filter noise out of the data thereby maintaining that only reasonable data points are used to train and predict the network flows. With the decision steps, the proposed model learns to adapt to specific characteristics of the data, hence, is able to improve the detection rate on network flows. With the reduced complexity that is achieved with the sparse feature representation, our TabNet-IDS is more efficient to provide a timely response in the detection and classification of the traffics.

**Fig 11:**
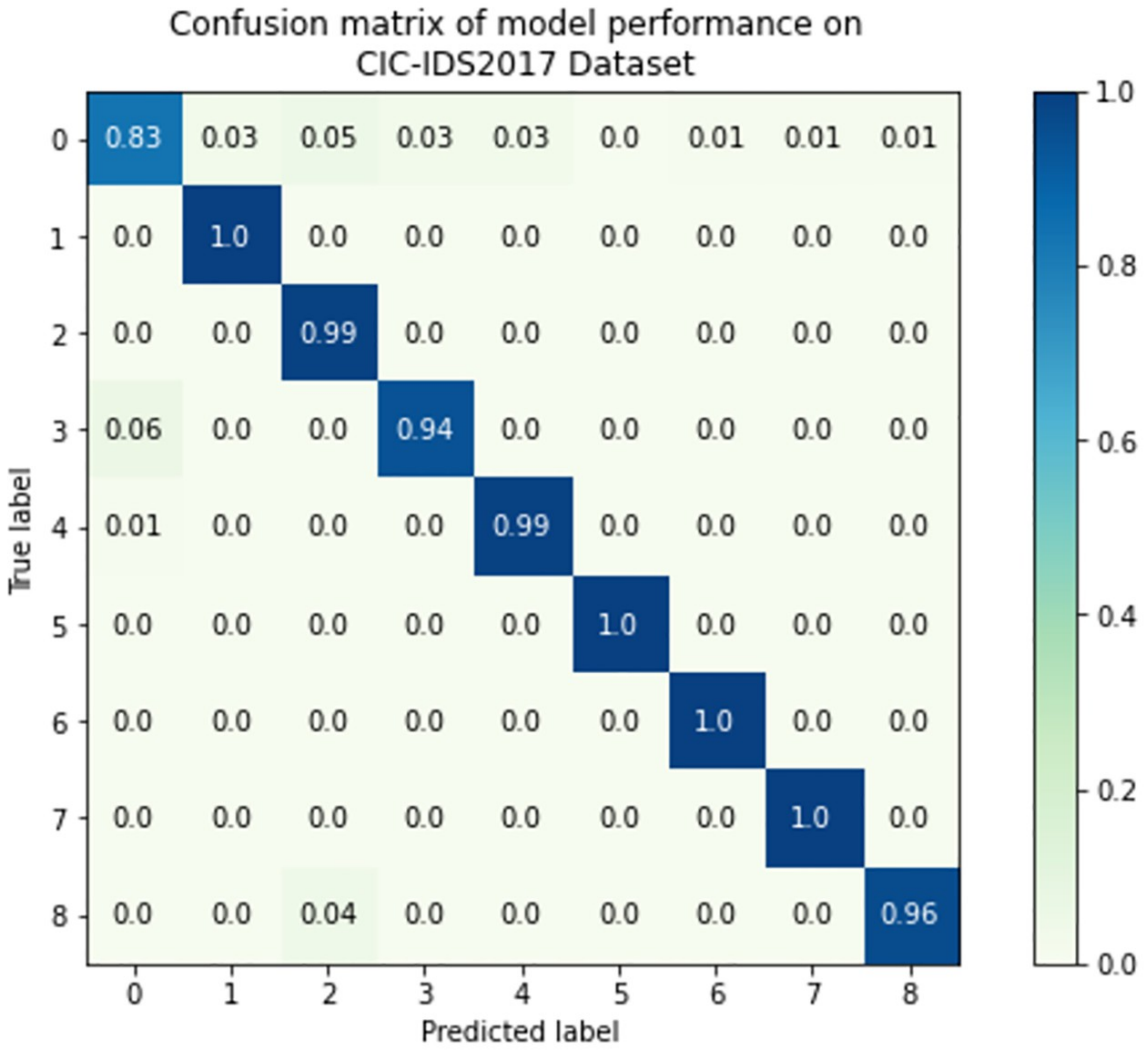
Confusion matrix of the model performance on the CIC-IDS2017 dataset. The encoded label is 0: Benign, 1: Botnet, 2: Brute force, 3: DDoS, 4: DoS, 5: Heartbleed, 6: Infiltration, 7: PortScan, 8: Web attacks.

**Fig 12:**
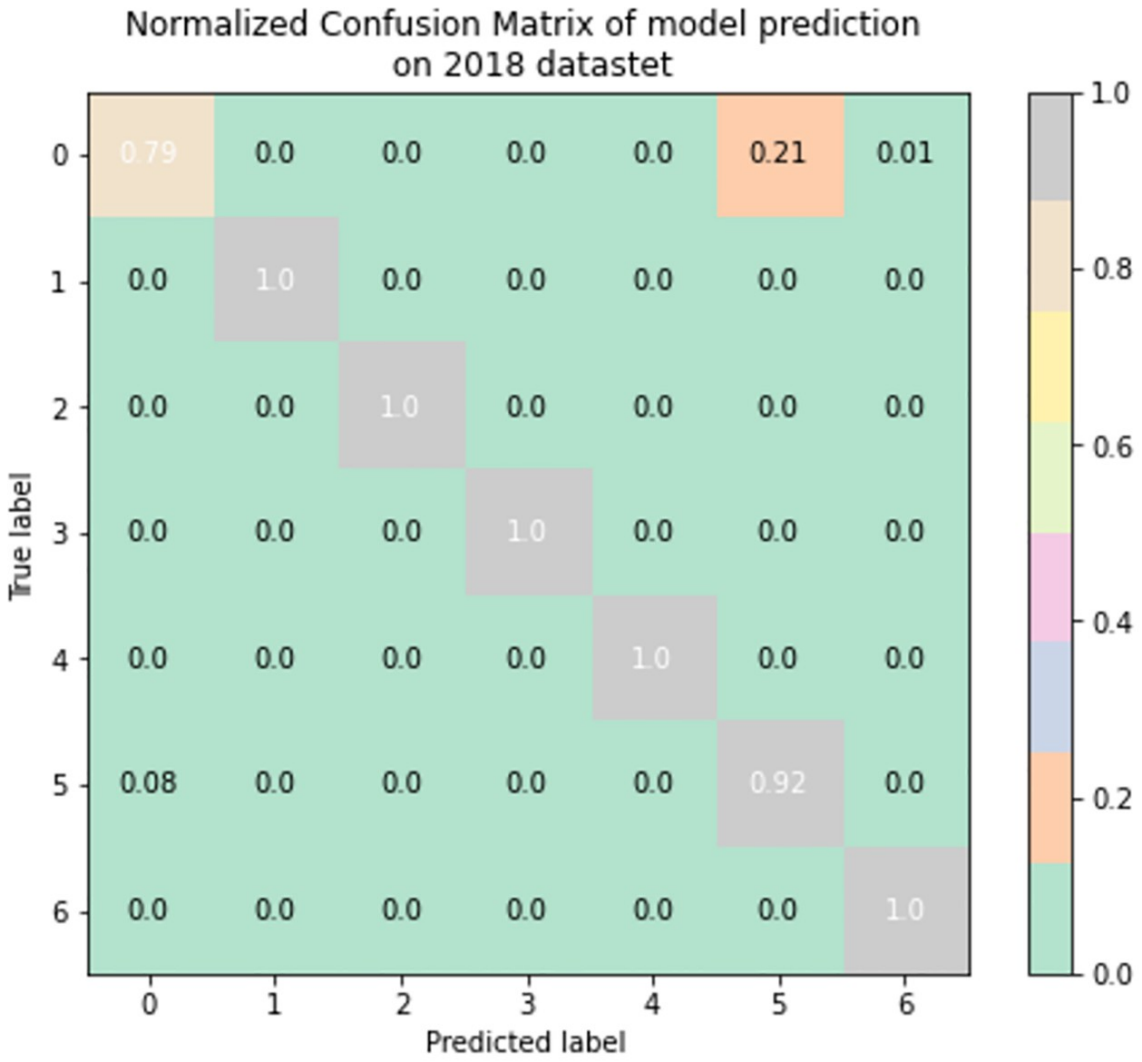
Confusion matrix of the model performance on the CSE-CICIDS2018 dataset with each label encoded as 0: Benign, 1: Bot, 2: DoS, 3: DDoS, 4: Brute force, 5: Infiltration, 6: Web Attacks.

**Fig 13:**
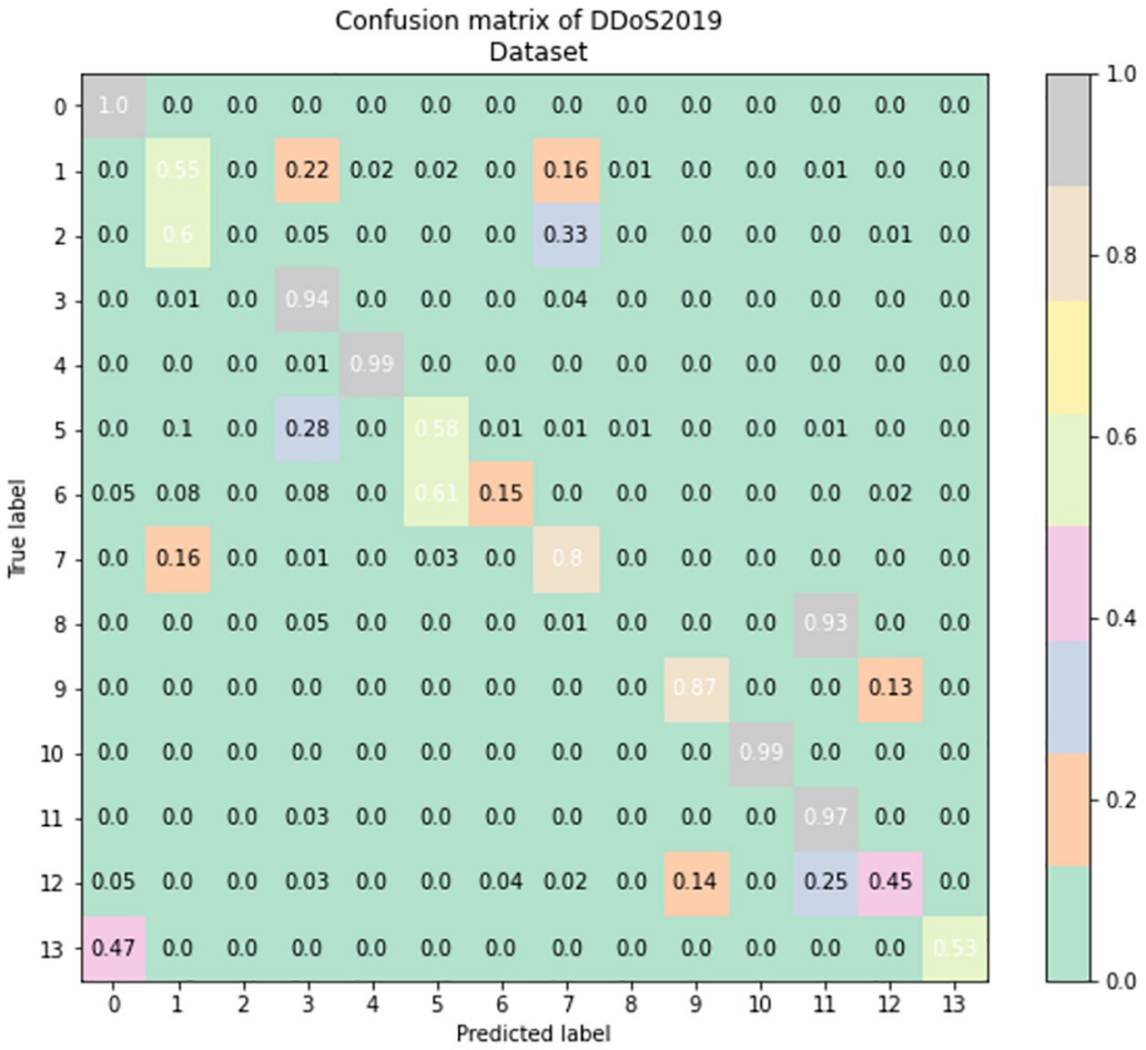
Confusion matrix of the model performance on the CIC-DDoS2019 dataset where each of the labels is encoded as 0: BENIGN, 1: DNS, 2: LDAP, 3: MSSQL, 4: NTP, 5: NetBIOS, 6: Portmap, 7: SNMP, 8: SSDP, 9: Syn, 10: TFTP, 11: UDP, 12: UDPLag, 13: WebDDoS.

To better understand the effectiveness of the TabNet-IDS model in categorizing each of the network flows, we evaluate the confusion matrix which depicts the connection between the actual values and the anticipated values in the dataset. These confusion matrices are shown in Figs 11–13, respectively, for the CIC-IDS2017, CSE-CICIDS2018, and CIC-DDoS2019 datasets. The normalized confusion matrices show that the proposed model can identify each of the attack vectors as well as the normal traffic in the datasets in all the scenarios involving multi-class network profile detection and classification. There are 9, 7, and 14 different classes in the CIC-IDS2017, CSE-CICIDS2018, and CIC-DDoS2019 that the model is built to identify. The TabNet-IDS shows good performance in each of these scenarios in accurately classifying the network flows with reduced False Positives.

### Performance comparison with related works

Considering that several ML and DL models already exist that have been implemented using different algorithms, it is crucial to compare their performance with our proposed TabNet-IDS model. This is aimed at highlighting the unique features and advantages of our model over existing ones. While accuracy is a key factor in evaluating the relevance of ML or DL model, explainability and interoperability are critical in providing insights into how the model makes its predictions. This is one of the primary focuses of our study. Explainability helps to provide more information on the reasons why specific features of the dataset determine the predictive results of the model. This, in turn, helps to increase the model’s transparency, making it easier to understand, interpret, and trust its predictions. As shown in Table 6, all deep learning-based models presented exhibited similar behaviors with different approaches. The evaluation metrics used showed results for accuracy, recall, and precision with the same range. Among the related works presented, only our proposed study implemented Matthew’s correlation coefficient (MCC) as an evaluation metric. This metric provides important information about the true positive, false positive, true negative, and false negative values in the prediction unlike the accuracy, precision, and recall. It is relevant to note that the implementation of the models used for comparison purposes was carried out on different datasets. The Tab-SRU proposed in [44] used the TabNet architecture for its implementation, and showed a high-performance accuracy as well as our results. While Chen et al [44] performed an experiment on the UKM-IDS20 and UNSW-NB15, we experimented on three more recent datasets. By incorporating explainability techniques into our proposed TabNet-IDS model, we provide detailed insights into the decision-making process of the model, making it easier to identify and address any biases or errors that might arise. As a result, we can improve the model’s overall accuracy and effectiveness in identifying network intrusions and enhancing the security of IoT devices.

**Table 6:** Comparison of the Performance of our proposed model and other models that implemented similar algorithms.

References	Method used	Accuracy (%)	Precision (%)	Recall (%)	F-Score (%)	MCC (%)
Yang et al [42]	BiLSTM	96.15	98.32	96.94	97.63	-
Singh et al [43]	GRU	89.97	88.32	97.94	92.84	-
Chen et al [44]	Tab-SRU	99.23	99.04	99.64	99.34	-
Yin et al [45]	RNN	88.93	87.76	92.51	90.07	-
Proposed Method A	TabNet-IDS (CIC-IDS2017)	97.03	97.10	97.02	96.97	96.97
Proposed Method B	TabNet-IDS (CSE-CICIDS2018)	95.58	95.69	95.59	95.55	95.55
Proposed method C	TabNet-IDS (CIC-DDoS2019)	98.51	98.50	98.40	98.44	97.52

## Conclusion

Over time, ML and DL algorithms have demonstrated high capabilities for providing enhanced network profile detection and classification, thereby ensuring that packets flowing through a network are reliable and secure. Due to the high dependence of IoT systems on the internet and sometimes delay in providing regular updates, they appear to be highly vulnerable to various forms of exploits. To ensure a more secure IoT ecosystem, ML and DL algorithms are mostly used. In this study, we investigated the application of the DL algorithm that is specifically targeted at tabular data, as most network profiles are extracted in tabular form. This aspect of ML and DL tasks has been under-explored and needs more attention. We experimented with the proposed model architecture on three different general-purpose datasets, including the CIC-IDS 2017, CSE-CICIDS 2018, and CIC-DDoS 2019. The TabNet-IDS model demonstrated a high detection rate and reduced the false alarm rate for detecting and classifying the network profile in a multi-class problem. The algorithm proposed in this study contributes to knowledge in the IDS domain by providing explanations for the choice of the features used in the predictive decision at each step of the training without requiring a third-party framework such as SHAPEly and LIME for interpretations, hence resulting in a lightweight IDS model. Due to the large parameter space in the TabNet algorithm, we performed hyper-parameter tuning to obtain the most relevant parameters and values that best describe the model’s performance while maintaining high accuracy. The model achieved an accuracy of 97%, 95%, and 98% on the CIC-IDS2017, CSE-CICIDS2018, and CIC-DDoS2019. Although the model shows competitive performances and provides interpretability, its varying behavior on different datasets is an acknowledged limitation. To address this, we intend to explore and investigate the model performance on additional datasets. We believe that by analyzing the model’s performance on a wider range of datasets, we can better understand the variations and make necessary adjustments to better improve the overall efficiency; and validate the model’s ability to generalize on unseen data for real-world applications.
